# Long-Term Dietary Taurine Lowers Plasma Levels of Cholesterol and Bile Acids

**DOI:** 10.3390/ijms23031793

**Published:** 2022-02-04

**Authors:** Ryoma Tagawa, Masaki Kobayashi, Misako Sakurai, Maho Yoshida, Hiroki Kaneko, Yuhei Mizunoe, Yuka Nozaki, Naoyuki Okita, Yuka Sudo, Yoshikazu Higami

**Affiliations:** 1 Department of Medicinal and Life Sciences, Faculty of Pharmaceutical Sciences, Tokyo University of Science, Chiba 278-8510, Japan; 3b13648@alumni.tus.ac.jp (R.T.); 3b18536@alumni.tus.ac.jp (M.S.); 3b17667@alumni.tus.ac.jp (M.Y.); 3a11019@alumni.tus.ac.jp (H.K.); nozaki@rs.tus.ac.jp (Y.N.); yukafeatkoro@me.com (Y.S.); 2 Department of Internal Medicine Endocrinology and Metabolism, Faculty of Medicine, University of Tsukuba, Tsukuba 305-8575, Japan; ymizunoe@md.tsukuba.ac.jp; 3 Division of Pathological Biochemistry, Faculty of Pharmaceutical Sciences, Sanyo-Onoda City University, Yamaguchi 756-0884, Japan; nokita7@rs.tusy.ac.jp; 4 Division of Integrated Research, Research Institute for Biomedical Sciences, Tokyo University of Science, Chiba 278-0022, Japan

**Keywords:** taurine, cholesterol, bile acid, FGF21, ERK, CYP7A1

## Abstract

Cholesterol is an essential lipid in vertebrates, but excess blood cholesterol promotes atherosclerosis. In the liver, cholesterol is metabolized to bile acids by cytochrome P450, family 7, subfamily a, polypeptide 1 (CYP7A1), the transcription of which is negatively regulated by the ERK pathway. Fibroblast growth factor 21 (FGF21), a hepatokine, induces ERK phosphorylation and suppresses *Cyp7a1* transcription. Taurine, a sulfur-containing amino acid, reportedly promotes cholesterol metabolism and lowers blood and hepatic cholesterol levels. However, the influence of long-term feeding of taurine on cholesterol levels and metabolism remains unclear. Here, to evaluate the more chronic effects of taurine on cholesterol levels, we analyzed mice fed a taurine-rich diet for 14–16 weeks. Long-term feeding of taurine lowered plasma cholesterol and bile acids without significantly changing other metabolic parameters, but hardly affected these levels in the liver. Moreover, taurine upregulated *Cyp7a1* levels, while downregulated phosphorylated ERK and *Fgf21* levels in the liver. Likewise, taurine-treated Hepa1-6 cells, a mouse hepatocyte line, exhibited downregulated *Fgf21* levels and upregulated promoter activity of *Cyp7a1*. These results indicate that taurine promotes cholesterol metabolism by suppressing the FGF21/ERK pathway followed by upregulating *Cyp7a1* expression. Collectively, this study shows that long-term feeding of taurine lowers both plasma cholesterol and bile acids, reinforcing that taurine effectively prevents hypercholesterolemia.

## 1. Introduction

Cholesterol is an essential lipid for lipid bilayers and biosynthesis of steroid hormones. However, obese patients often show a high blood cholesterol level, which promotes atherosclerosis [1,2]. The blood levels of cholesterol are strictly regulated mainly by the liver, which releases cholesterol in the form of very low-density lipoprotein (VLDL) and takes in cholesterol through the low-density-lipoprotein (LDL) receptor [3]. Cholesterol is converted to bile acids, including cholic acids and deoxycholic acids, in the liver; steroid hormones, including sex hormones, in the testes or ovaries; and glucocorticoid, in the adrenal cortex. Interestingly, these metabolites modulate the synthesis of each other. Specifically, estradiol and progesterone reportedly regulate the synthesis of bile acids in hepatocytes, while bile acids are involved in various steps of the synthetic pathway of glucocorticoids [4,5,6]. Bile acids are secreted to the duodenum, where they support the activity of lipase by forming micelles with lipids during digestion [7]. In fact, the deletion of sterol 12-alpha-hydroxylase, an enzyme involved in the synthesis of bile acids, causes lowered levels of bile acid and impaired absorption of lipids in mice [8,9]. Most bile acids are reabsorbed in the small intestinal tract and retransported to the liver [10]. Bile acids are also known as signaling metabolites, which regulate lipid and glucose metabolism [11,12,13,14]. In addition, bile acids contribute to energy expenditure associated with thermogenesis in brown adipose tissue [15]. On the basis of the above, altered bile acid synthesis can affect systemic metabolism, as well as lipid digestion.

The metabolic pathway of cholesterol is classified into the classical pathway responsible for bile acid synthesis in the liver, and the alternative pathway mainly responsible for the synthesis of bile acids and steroid hormones in macrophages or the adrenal cortex [16]. Liver expresses all of the enzymes involved in the classical pathway [16]. Among these enzymes, cytochrome P450, family 7, subfamily a, polypeptide 1 (CYP7A1) is the rate-limiting enzyme of the conversion of cholesterol into bile acids [17,18,19,20,21,22]. Humans with CYP7A1 loss-of-function mutations exhibit high levels of circulating LDL cholesterol [23]. The expression of *Cyp7a1* is reportedly regulated by bile acids as follows. Bile acids activate farnesoid X receptor (FXR), a nuclear receptor, and induce its downstream pathways, including small heterodimer partner (SHP) and liver receptor homolog-1 (LRH-1), thereby suppressing the expression of *Cyp7a1* in the liver. To explain this signal in brief, the binding of bile acids to FXR in the liver transcriptionally induces SHP, which inhibits the transcriptional activity of LHR-1, leading to suppressed expression of *Cyp7a1* [24]. In addition to this, bile acids also activate FXR and promote the secretion of fibroblast growth factor 15/19 (FGF15/19) in the ileum. Circulating FGF15/19 binds to the FGF receptor 4 (FGFR4)–Klotho beta (KLB) complex, which is abundant in the liver, and suppresses expression of the *Cyp7a1* gene. It has also been reported that the members of MAPK cascades, such as extracellular signal-regulated kinase (ERK) and c-Jun N-terminal kinase (JNK), downregulate the expression of *Cyp7a1* [25].

Fibroblast growth factor 21 (FGF21) is a hepatokine that regulates lipid and glucose metabolism [26,27]. After secretion from the liver, FGF21 mainly binds to the FGF receptor 1 (FGFR1) and beta-klotho (KLB) receptor complex in target tissues, such as white adipose tissue (WAT) and muscle, which in turn activates the downstream signals [20,28,29]. FGF21 also protects against hepatic insulin resistance and steatosis in obese mice and regulates glycogen synthesis and ketone body production in the liver [30,31,32,33]. Chen et al. demonstrated that, in primary hepatocytes and several rodent models, FGF21 suppresses expression of the *Cyp7a1* gene via the induction of ERK phosphorylation [34]. FGF21 is also a well-known stress-responsive cytokine [35]. In fact, previous studies showed that FGF21 levels are elevated in the blood of obese individuals and liver in patients with nonalcoholic fatty liver disease [36,37].

Taurine (NH_3_^+^-CH_2_-CH_2_-SO_3_^−^), a sulfur-containing amino acid that is not used for protein synthesis and exists as a free amino acid, is endogenously made from cysteine or methionine, or exogenously provided by the diet [38,39]. Taurine is known to regulate oxidative stress, osmotic pressure, intracellular calcium concentration, and autophagy [40,41,42,43]. Previous studies have shown that taurine regulates metabolic pathways of glucose and lipids. For example, taurine improves hyperglycemia in diabetic model animals by modifying glucagon activity, insulin sensitivity, and insulin secretion [44]. Taurine also inhibits fatty acid biosynthesis and enhances the catabolism of triglycerides in the liver, thereby preventing high-fat-diet-induced hepatic steatosis [45]. In addition to the above, many findings support the effects of taurine on the levels of cholesterol and its metabolism [46]. Actually, a taurine-supplemented diet reportedly promotes cholesterol degradation, thereby lowering serum and hepatic cholesterol levels [10,47]. Moreover, taurine-supplemented water is reported to increase hepatic LDL receptors and allow its internalization and degradation [48]. Although accumulated evidence has shown that taurine can lower plasma cholesterol levels, many of these studies evaluated its effects using animals fed taurine for a relatively short period (e.g., 2 weeks) [49]. Hence, current findings fail to sufficiently demonstrate the continuous involvement of taurine in cholesterol metabolism. In the present study, to evaluate the more chronic effects of taurine on cholesterol metabolism, we analyzed cholesterol-related metabolic parameters and pathways in mice fed taurine for a long period.

## 2. Results

### 2.1. Taurine Lowered Plasma Levels of Cholesterol and Bile Acids

**Figure 1:**
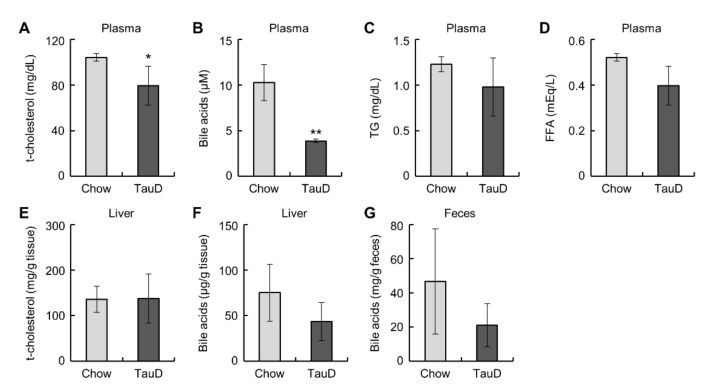
Taurine lowered plasma cholesterol and bile acids: Plasma total cholesterol (t-cholesterol) (n = 5) (**A**), bile acids (n = 4) (**B**), triglyceride (TG) (n = 5) (**C**), non-esterified fatty acids (NEFA) (n = 5) (**D**), t-cholesterol (n = 10–11) (**E**), and bile acids (n = 5) (**F**) in the liver, and bile acids (**G**) in feces (n = 4) of Chow and TauD groups. Values represent means ± SD. Differences between values were statistically evaluated by Student’s *t*-test. * *p* < 0.05, ** *p* < 0.01 vs. Chow.

Taurine did not affect body weight, food intake, or the weight of metabolic tissues, such as the liver, epididymal WAT (eWAT), and inguinal subcutaneous WAT (iWAT) (Figure S1A–F, see Supplementary Materials). To evaluate the basal metabolic rate and glucose metabolism in the whole body, we calculated the respiratory exchange ratio (RER) from respiration measurements and performed a glucose tolerance test (GTT) and an insulin tolerance test (ITT) in control diet (Chow) and taurine-supplemented diet (TauD) groups. The results showed that these parameters were not significantly changed (Figure S2A–E). Likewise, basal plasma glucose levels also showed no significant difference between the two groups (Figure S2F). These results indicate that taurine exerted little influence on whole-body metabolism in this model. Subsequently, we measured plasma lipid parameters, cholesterol, bile acids, triglycerides (TG), and non-esterified fatty acids (NEFA). The TauD group showed lower plasma levels of cholesterol and total bile acids, but no significant change in TG and NEFA levels (Figure 1A–D). In contrast, the levels of cholesterol and bile acids in the liver and bile acids in feces were unchanged between the Chow and TauD groups (Figure 1E–G). These findings suggest that taurine strongly induced the catabolism of cholesterol and bile acid transported into the liver, resulting in their lowered plasma levels.

### 2.2. Taurine Induced the Expression of the Cyp7a1 Gene and Suppressed the FGF21-ERK Signal in the Liver

**Figure 2:**
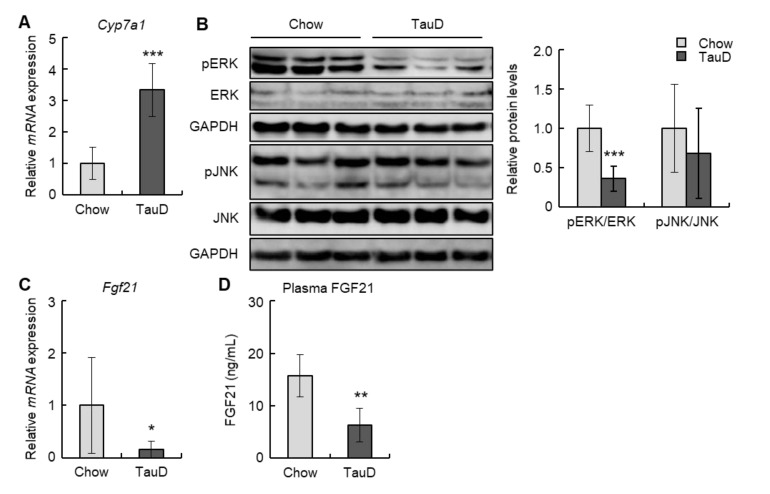
Taurine downregulated FGF21/ERK pathway and upregulated *Cyp7a1* transcription in the liver: (**A**) The expression of *Cyp7a1* mRNA in the liver was analyzed by real-time RT-PCR (n = 6). (**B**) Total protein extracted from the liver was analyzed by immunoblotting using the shown antibodies (n = 6). The left panels show representative images. The right graphs show quantitative data. GAPDH was used as a loading control. (**C**) The expression of *Fgf21* mRNA in the liver was analyzed by real-time RT-PCR (n = 6). (**D**) Plasma FGF21 was quantified using ELISA (n = 6). Data of real-time RT-PCR were normalized to *Tbp* levels. Values represent means ± SD. Differences between values were statistically evaluated by Student’s *t*-test. * *p* < 0.05, ** *p* < 0.01, *** *p* < 0.001 vs. Chow.

To evaluate the impact of taurine on cholesterol metabolism, we initially analyzed the expression of the *Cyp7a1* gene, which encodes a rate-limiting enzyme converting cholesterol into bile acid, in the liver. In the TauD group, *Cyp7a1* levels were markedly upregulated (Figure 2A). Next, we examined the levels of phosphorylated ERK and JNK, negative regulators of *Cyp7a1*, in the liver. Although the phosphorylation levels of JNK were unchanged, those of ERK were decreased in the TauD group (Figure 2B). Subsequently, we analyzed the hepatic levels of *Fgf21*, an upstream regulator of ERK. The results showed that taurine downregulated the hepatic levels of *Fgf21* (Figure 2C). In agreement with this, plasma FGF21 levels were also decreased in the TauD group (Figure 2D). These findings suggest that taurine upregulated expression of the *Cyp7a1* gene by suppressing the FGF21/ERK pathway in the liver.

### 2.3. Taurine Suppressed the Expression of FGF21 and Enhanced the Activity of Cyp7a1 Promoter in Hepa1-6 Cells

**Figure 3:**
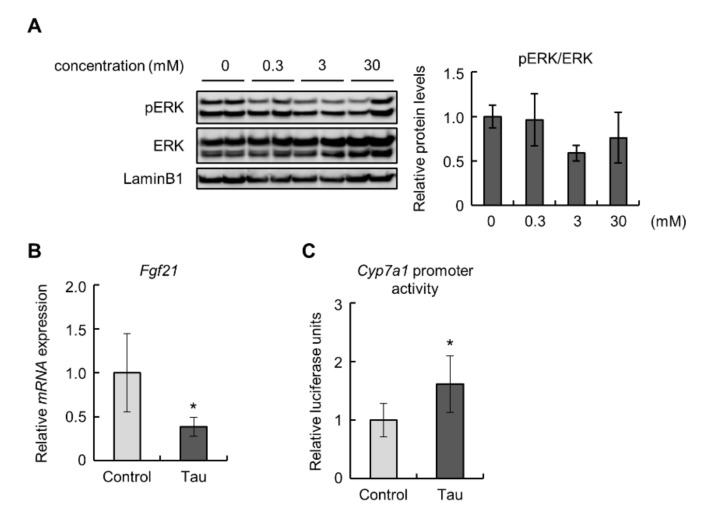
Taurine downregulated *Fdf21* levels and induced *Cyp7a1* promoter activity in Hepa1-6 cells: (**A**) Total protein extracted from Hepa1-6 cells treated with the indicated concentrations of taurine for 24 h was analyzed by immunoblotting using the shown antibodies (n = 4). The left panels show representative images. The right graphs show quantitative data. LaminB1 was used as a loading control. (**B**) The expression of *Fgf21* mRNA in 10 mM taurine-treated Hepa1-6 cells (Tau) was analyzed by real-time RT-PCR. Data were normalized to *Rps18* (n = 6). (**C**) Luciferase assay of *Cyp7a1* in 10 mM taurine-treated Hepa1-6 cells (Tau). Relative luminescent units (RLU) were calculated as shown in the Materials and Methods section (n = 4). Values represent means ± SD. Differences between values were statistically evaluated by Dunnett’s test (**A**) or Student’s *t*-test (**B**,**C**). * *p* < 0.05 vs. Control.

To confirm the effects of taurine on the FGF21/ERK pathway in the liver, we examined Hepa1-6 cells treated with taurine. The phosphorylation levels of ERK were decreased, but not significantly, in 3 mM taurine-treated Hepa1-6 cells (Figure 3A). Similar to the results obtained from in vivo experiments, taurine downregulated the expression of the *Fgf21* gene (Figure 3B). Next, we performed a luciferase assay to examine the promoter activity of *Cyp7a1* in Hepa1-6 cells treated with taurine because the detected expression levels of *Cyp7a1* were insufficient for quantitative evaluation. The results showed that taurine enhanced *Cyp7a1* promoter activity (Figure 3C). These findings suggest that taurine suppressed the expression of FGF21 and induced *Cyp7a1* transcription.

## 3. Discussion

In the present study, we demonstrated that long-term feeding of taurine lowered plasma cholesterol and suppressed the FGF21/ERK pathway in mice. The former effect is consistent with previous articles reporting that taurine prevents hypercholesterolemia [47,48]. Murakami et al. showed that taurine enhances the uptake of LDL cholesterol into the liver, resulting in lowered plasma cholesterol [48]. Given our results showing that taurine did not alter cholesterol levels in the liver (Figure 1E,F), taurine is likely to induce the catabolism of cholesterol transported into the liver, as well as its uptake. Moreover, taurine did not change the contents of bile acids, a catabolic product of cholesterol, in the liver and feces (Figure 1F,G), implying that taurine may promote the transport of produced bile acids into the blood circulation. However, plasma levels of bile acids were rather reduced in the taurine-fed group (Figure 1B). In general, taurine conjugates with bile acids, thereby promoting bile excretion by increasing their water solubility into bile [50,51]. In fact, Miyazaki et al. reported that a taurine-deficient diet decreased bile acid excretion into bile in cats, which have a low ability to biosynthesize taurine [52]. Therefore, it is conceivable that taurine can enhance not only the catabolism of cholesterol but also the enterohepatic circulation of bile acids via conjugation, leading to lowered plasma levels of cholesterol and bile acids.

Taurine lowered the levels of plasma FGF21 and *Fgf21* mRNA in the liver and Hepa1-6 cells (Figure 2C,D and Figure 3B). This result is partially supported by a previous report describing that taurine suppressed the level of FGF21 in the liver of cafeteria-diet-fed rats [53]. Taurine also significantly enhanced *Cyp7a1* transcription and suppressed the phosphorylation of ERK in the liver (Figure 2A,B). FGF21 has been proven to suppress *Cyp7a1* mRNA expression in hepatocytes [20,34]. In contrast, Keinicke et al. reported that FGF21 administration upregulated *Cyp7a1* mRNA expression in the liver of mice with diet-induced obesity [54]. Zhang et al. also demonstrated that adeno-associated virus-mediated overexpression of *Fgf21* in mice increased liver expression of *Cyp7a1* [55]. However, it has also been reported that FGF21 administration exerts no influence on this expression [56,57]. These controversial findings suggest that differences in experimental conditions can markedly affect the relationship between FGF21 and *Cyp7a1* expression. Moreover, although the FGF21-ERK signal generally requires the binding of FGF21 to FGFR1, FGFR1 is hardly expressed in the liver [13]. Hence, the above effects of FGF21 in hepatocytes can be mediated by other FGFRs, for example, FGFR3, which is reportedly expressed in the liver and bound by FGF21 [58,59]. 

*Cyp7a1* is positively regulated by hepatocyte nuclear factor 4α (HNF4α), whose activity is known to be suppressed by ERK via the induction of its phosphorylation and extranuclear transport [60,61]. In Hepa1-6 cells, taurine activated *Cyp7a1* transcription (Figure 3C). The *Cyp7a1* promoter region used in this study contains an HNF4α-binding site [25]. Furthermore, taurine reportedly increases the level of HNF4α protein and its transcriptional activity for *Cyp7a1* [62]. However, in our analysis, taurine failed to significantly change the nuclear amount of HNF4α in Hepa1-6 cells despite downregulated *Cyp7a1* (Figure S5). The transcriptional activity of HNF4α requires not only its nuclear localization but also homodimer formation [63]. ERK has been demonstrated to inhibit homodimer formation by the phosphorylation of HNF4α [60]. Based on these findings, the unchanged nuclear HNF4α in taurine-treated Hepa1-6 cells implies that taurine may maintain homodimer formation of HNF4α via suppressed ERK activity, resulting in upregulated transcription of *Cyp7a1*.

In addition to the above pathways, the transcription of *Cyp7a1* is regulated by a negative feedback loop containing the following members of the nuclear receptor family: FXR, SHP, and LRH-1 [24]. Our results showed that taurine exerted no effect on the mRNA levels of *Fxr*, *Shp*, and *Lrh-1* in the liver, indicating that taurine-induced increase in *Cyp7a1* expression is not highly relevant to this signal pathway (Figure S3). Transcriptional regulation of *Cyp7a1* also includes the FGF15/19-FGFR4-KLB signal. Feeding-induced bile acids increase *Fgf15/19* expression in the ileum, thereby elevating serum FGF15/19 levels [64]. The binding of FGF15/19 to FGFR4 suppresses the expression of *Cyp7a1* in the liver [24]. In our analysis, taurine did not alter the levels of *Fgf15/19* in the ileum (Figure S4), supported by the result of the amount of bile acids in feces remaining unchanged (Figure 1G). This is inconsistent with a recent study showing that taurine decreased *Fgf15* in the ileum [65]. This discrepancy may be due to differences in the duration of feeding of taurine. Taking these findings together, it is conceivable that long-term feeding of taurine induces an increase in *Cyp7a1* expression and a reduction in plasma cholesterol levels, which may be mainly attributed to the FGF21-ERK signal.

Apart from CYP7A1, cholesterol is known to be metabolized by 11β-hydroxysteroid dehydrogenase 1 (11B-HSD1), an enzyme that interconverts cortisone and cortisol, thereby regenerating active glucocorticoids [66]. To be exact, 7-oxocholesterol is a substrate of 11B-HSD1 [67,68]. Taurine reportedly prevents glucocorticoid-induced osteonecrosis and muscle atrophy, which implies that taurine may affect the activity of 11B-HSD1 and suppress glucocorticoid-induced physiological reactions [69,70]. Despite no direct evidence of the relationship between taurine and 11B-HSD, taurine may contribute to cholesterol metabolism via not only increased expression of *Cyp7a1,* but also regulation of the activity of 11B-HSD1.

**Figure 4:**
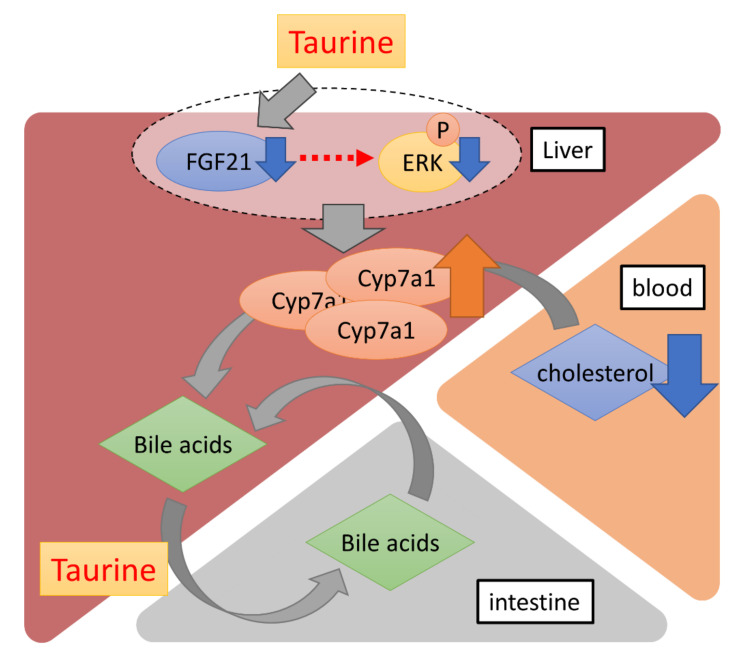
A schematic diagram of the effects of long-term dietary taurine. Taurine upregulates the transcriptional activity of *Cyp7a1* by suppressing FGF21 production in the liver. Bile acids are converted from blood cholesterol by CYP7A1 and more efficiently enter the enterohepatic circulation via taurine conjugation.

In conclusion, our data showed that long-term dietary taurine lowered plasma cholesterol and bile acids, probably by promoting cholesterol metabolism via the induction of CYP7A1. Additionally, taurine suppressed the production of FGF21 in the liver (Figure 4). Although FGF21 is known to contribute to systemic glucose metabolism as mentioned above, there was no difference in glucose tolerance between the Chow and TauD groups. Thus, a taurine-induced decrease in FGF21 could locally affect cholesterol metabolism in the liver in an autocrine or paracrine manner. Given that bile acids are metabolites of cholesterol, a taurine-induced decrease in bile acids with induced-cholesterol metabolism can reflect more efficient prevention of hypercholesterolemia. Taurine is easily taken in from seafood diets and available supplements in daily life. Furthermore, few side effects of taurine have been reported, which is consistent with our results that TauD groups exhibited no observable side effects. These features confer advantages for the use of taurine in continuously lowering blood cholesterol. 

## 4. Materials and Methods

### 4.1. Animal Experiments

Animal experiments were approved by the Ethics Review Committee for Animal Experimentation at Tokyo University of Science (approval numbers: Y18058, Y19055, and Y20044). Mice were maintained under specific-pathogen-free conditions at 23 °C and a 12 h light/dark cycle in the animal facility at the Faculty of Pharmaceutical Sciences, Tokyo University of Science. They had free access to water and were fed a Charles River Formula-1 (CRF-1) diet (21.9% crude protein, 5.4% crude fat, and 2.9% crude fiber; Oriental Yeast, Tokyo, Japan) or CRF-1 supplemented with 5% taurine (Fujifilm Wako Pure Chemical, Osaka, Japan). Male 3-week-old C57BL/6J mice were purchased from CLEA Japan (Tokyo, Japan) and divided into two groups: one was a Chow group fed the CRF-1 diet, while the other was a TauD group fed CRF-1 supplemented with taurine. During rearing, body weight and food intake of the two groups were continuously measured. At 18 weeks of age, resting oxygen consumption and resting carbon dioxide output were measured using an MK-5000RQ (Muromachi Kikai Co., Ltd., Tokyo, Japan) and the RER was calculated. At this time, a GTT was performed. Two days later, an ITT was performed. At 20 weeks of age, body weight of the two groups was measured after euthanasia with isoflurane anesthesia (Mylan, Canonsburg, PA, USA). The last third of the harvested small intestine was separated as the ileum. Subsequently, the ileum was incised vertically and its mucosa was scraped and removed. The other tissues were collected, washed using phosphate-buffered saline (PBS), and weighed. Then, tissue samples were minced, snap-frozen in liquid nitrogen, and stored at −80 °C until use. Blood samples were mixed with 100 mM EDTA and centrifuged at 2500× *g* for 10 min at 4 °C. The supernatant was collected as plasma samples and stored at −80 °C until analysis.

### 4.2. Plasma Biochemical Analysis

Plasma glucose, TG, NEFA, cholesterol, total bile acid, and FGF21 levels were measured using Autokit Glucose CII (Fujifilm Wako Pure Chemical, Osaka, Japan), LabAssay Triglyceride (Fujifilm Wako Pure Chemical, Osaka, Japan), LabAssay NEFA (Fujifilm Wako Pure Chemical, Osaka, Japan), LabAssay Cholesterol (Fujifilm Wako Pure Chemical, Osaka, Japan), Total Bile Acid Assay Kit (Cell Biolabs, San Diego, CA, USA), and Mouse/Rat FGF21 Quantikine ELISA kit (R & D Systems, Minneapolis, MN, USA), respectively. All assays were performed in accordance with the manufacturers’ protocols.

### 4.3. Quantitative RT-PCR

Total RNA was extracted from frozen liver, ileum, or cells using ISOGEN II (Nippon Gene, Toyama, Japan), and reverse transcription was performed using ReverTra Ace qPCR RT Master Mix (Toyobo, Osaka, Japan). Quantitative PCR was performed using the CFX Connect™ Real Time System (Bio-Rad, Hercules, CA, USA) and Thunderbird SYBR qPCR Mix (Toyobo, Osaka, Japan), in accordance with the manufacturers’ protocols. Quantitative PCR data were processed using a standard curve method. TATA binding protein (*Tbp*) or Ribosomal protein S18 (*Rps18*) was used as a housekeeping gene. Sequences of the primers used for PCR are shown in Table 1.

### 4.4. Immunoblotting

Protein extraction and Western blotting were performed as described in our previous report [71]. Briefly, livers were homogenized in SDS sample buffer (50 mm Tris-HCl (pH 6.8), 2% SDS, 3 M urea, 6% glycerol), centrifuged at 12,000× *g* for 30 min at 4 °C, and the supernatant was boiled for 5 min. Cells were lysed in SDS sample buffer, boiled for 5 min, and sonicated. Lysates were subjected to SDS/PAGE and separated proteins were transferred to nitrocellulose membranes. Membranes were blocked with blocking solution (2.5% skim milk, 0.25% BSA in TTBS (25 mM Tris-HCl [pH 7.4], 140 mM NaCl, 2.5 mM KCl, 0.1% Tween-20)) for 60 min at room temperature and then probed with appropriate primary antibodies overnight at 4 °C. The anti-phospho ERK1 pT202/ERK2 pT185 (#4370), anti-ERK 1/2 (#9102), and anti-phospho JNK (#4668) antibodies were purchased from Cell Signaling Technology (Danvers, MA, USA); the anti-Glyceraldehyde 3-phosphate dehydrogenase (GAPDH) antibody (010-25521) was purchased from Fujifilm Wako Pure Chemical, Osaka, Japan; and the anti-JNK (sc-7345) and anti-Hepatocyte Nuclear Factor 4 alpha (HNF4α) antibodies (sc-6556) were purchased from Santa Cruz Biotechnology (Dallas, TX, USA). After probing with primary antibodies, membranes were incubated with appropriate secondary antibodies for 60 min at room temperature. The used secondary antibodies were horseradish peroxidase-conjugated F(ab’)2 fragment of goat anti-mouse IgG or anti-rabbit IgG (Jackson ImmunoResearch, West Grove, PA, USA). Antibody-bound proteins were visualized using ImmunoStar LD Reagent (Fujifilm Wako Pure Chemical, Osaka, Japan) and a LAS3000 Image Analyzer (Fujifilm, Tokyo, Japan), and data were analyzed using multigauge software (Fujifilm, Tokyo, Japan).

### 4.5. Cell Culture and Treatment

Hepa1-6 cells were purchased from RIKEN Bioresource Center (Ibaraki, Japan). Hepa1-6 cells were maintained in Dulbecco’s Modified Eagle Medium (D-MEM) (High Glucose) (Fujifilm Wako Pure Chemical, Osaka, Japan) supplemented with 10% fetal bovine serum (FBS) (Capricorn Scientific, Ebsdorfergrund, Germany) and 1% penicillin/streptomycin (Sigma, MO, USA) under a humidified incubator with 5% CO_2_ at 37 °C. For the analysis, Hepa1-6 cells were treated with taurine (Fujifilm Wako Pure Chemical, Osaka, Japan) for 24 h and then collected. The applied concentration is shown in the legends of Figure 3 and Figure S5.

### 4.6. Luciferase Assay for Cyp7a1 Promoter Activity

The *Cyp7a1* promoter-driven firefly luciferase plasmid (pGL4.10-Cyp7a1) was generated as follows. The fragments of the *Cyp7a1* promoter region (−376/+32), as identified in a previous study [25], were amplified from rat genomic DNA using KOD FX Neo (Toyobo, Osaka, Japan) with the following primers: forward primer 5′-TTT CTC GAG TGG GAA GCT TCT GCC TGT TT-3′ and reverse primer 5′-CCC AGA TCT TGC AAA AGC AGG AAA ATT TCC AAA GGG G-3′. Underlined letters represent XhoI and BglII sites, respectively. The generated insert was digested with XhoI and BglII and then subcloned into pGL4.10 (Promega, Madison, WI, USA) digested with the same enzymes. The luciferase assay was performed using Dual-Luciferase Reporter Assay System (Promega, Madison, WI, USA), in accordance with the manufacturer’s protocol. In brief, 0.5–1.1 × 10^4^ Hepa1-6 cells/well were seeded on 96-well plates. After 24–48 h, cells were transfected with pGL4.10-Cyp7a1 and pGL4.74 (Promega, Madison, WI, USA), a plasmid coding HSV-TK promoter-driven *Renilla* luciferase, using TransIT-2020 Transfection Reagent (Takara, Shiga, Japan) and cultured for 24–72 h. Thereafter, luciferase activity in transfected cells was measured using the above-mentioned kit and an EnVision Multilabel Reader (PerkinElmer, Waltham, MA, USA). The data are shown as the ratio of firefly luminescence to *Renilla* luminescence (relative luminescent units: RLU).

### 4.7. Measurement of Fecal Bile Acids

Approximately 60 mg of feces was homogenized in 400 μL of cold PBS. The homogenates were centrifuged at 10,000× *g* for 10 min at 4 °C. Then, the supernatant was collected, and the bile acid levels were measured using Total Bile Acid Assay Kit (San Diego, CA, USA).

### 4.8. Isolation of Nuclear Fractions

Harvested cell pellets were suspended in buffer A (20 mM HEPES (pH 7.9), 3 mM MgCl_2_, 20 mM KCl, 0.68 M sucrose, 20% glycerol, and 1% Triton X-100) and incubated on ice for 10 min. Cells were disrupted by pipetting and the suspension was centrifuged at 1300× *g* for 5 min. After discarding the supernatant, the precipitate was resuspended in Wash buffer (20 mM HEPES (pH 7.9), 3 mM MgCl_2_, 20 mM KCl, 0.68 M sucrose, and 20% glycerol) and centrifuged at 1300× *g* for 4 min. The supernatant was discarded, and the precipitate was resuspended in Wash buffer and centrifuged again at 1300× *g* for 4 min. The precipitate was obtained as the nuclear fraction.

### 4.9. Statistical Analyses

All data are expressed as mean ± standard deviation (SD). Statistical significance was determined by Student’s *t*-test or Dunnett’s test. Differences were considered significant at *p* < 0.05.

## Figures and Tables

**Table 1:** List of primers for RT-PCR.

Genes	Forward (5′ to 3′)	Reverse (5′ to 3′)
*Cyp7a1*	AGCAACTAAACAACCTGCCAGTACTA	GTCCGGATATTCAAGGATGCA
*Fgf21*	GAAGCCCACCTGGAGATCAG	CAAAGTGAGGCGATCCATAGAG
*Fxr*	CCAACCTGGGTTTCTACCC	CACACAGCTCATCCCCTTT
*Shp*	CGATCCTCTTCAACCCAGATG	AGGGCTCCAAGACTTCACACA
*Lrh-1*	ACTGAGAAATTCGGACAGCTACTTC	AGGTAGTCTTCTGCCTGCTTGCT
*Fgfr4*	GACCAAACCAGCACCGTGGCTGTGAAGATG	GTTTCCCTTGGCGGCACATTCCACAATCAC
*Klb*	CACTGTGGGACACAACCTGA	CCAAGCACAGAGGACATGGA
*Fgf15*	ACCGCTCCTTCTTTGAAAC	TACATCCTCCACCATCCTGAAC
*Tbp*	CAGTACAGCAATCAACATCTCAGC	CAAGTTTACAGCCAAGATTCACG
*Rps18*	TGCGAGTACTCAACACCAACAT	CTTTCCTCAACACCACATGAGC

## Data Availability

Not applicable.

## Supplementary Materials

The following supporting information can be downloaded at: https://www.mdpi.com/article/10.3390/ijms23031793/s1.
