# Supplementary material for: Long-Term Dietary Taurine Lowers Plasma Levels of Cholesterol and Bile Acids
Source: Int J Mol Sci. 2022 Feb 4;23(3):1793. doi: 10.3390/ijms23031793 (PMC8836270; doi:10.3390/ijms23031793)
Supplement: Supplementary file 1 [file ijms-23-01793-s001.zip › ijms-1548844-supplementary.pdf]

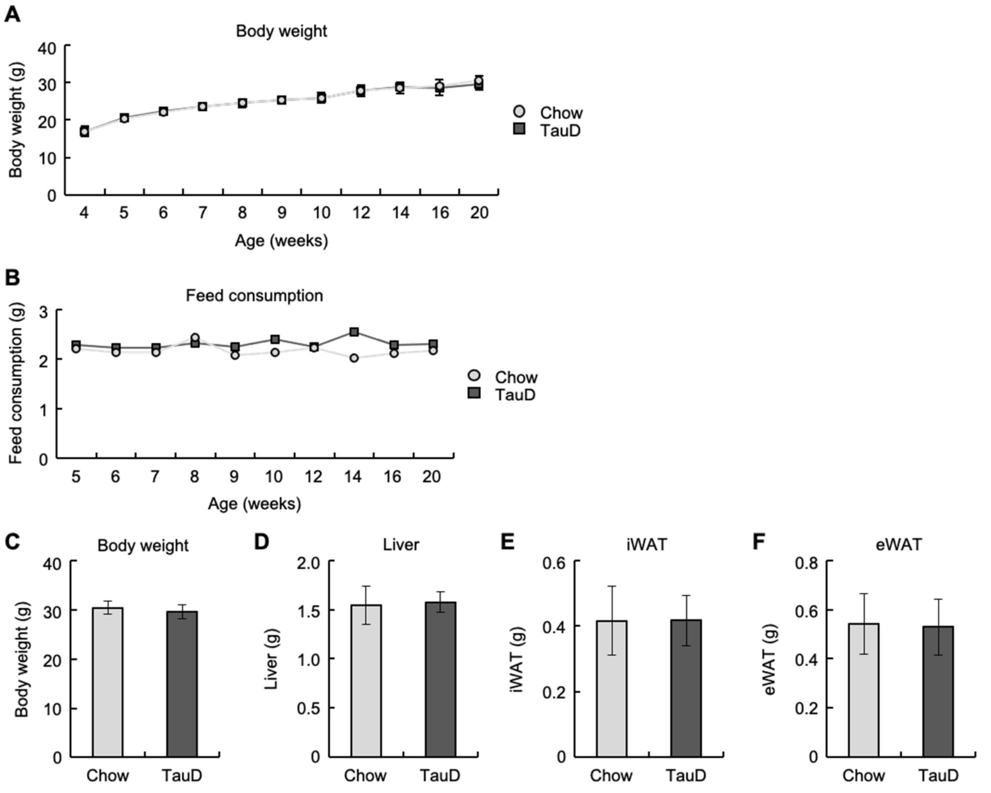

Figure S1. Basic data of Chow and TauD groups. (**A**, **B**) The time courses of body weight (**A**) and feed consumption (**B**) (n = 10–11). (**C**–**F**) The weights of body (**C**), liver (**D**), inguinal subcutaneous white adipose tissue (iWAT) (**E**), and epididymal white adipose tissue (eWAT) (**F**) at 20 weeks of age (n = 10–11). Values represent means ± SD.

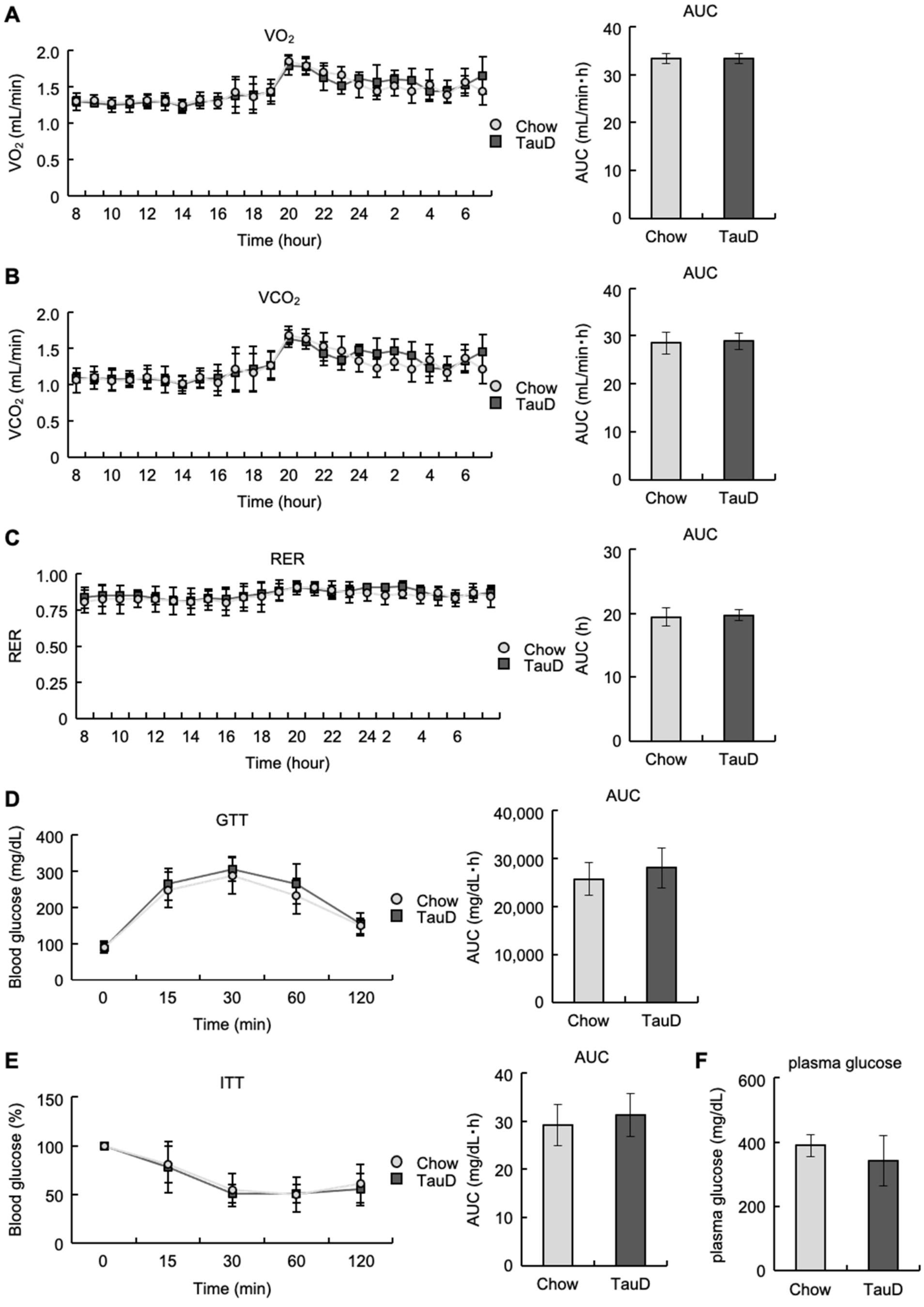

Figure S2. Respiratory metabolism and glucose metabolism of Chow and TauD groups. (**A**–**C**) VO_2 (O_2 consumption) (**A**), VCO_2 (CO_2 production) (**B**), and respiratory exchange ratio (RER=VCO_2/VO_2) (**C**) during light and dark phases at 18 weeks of age (n=6). (**D**, **E**) Glucose tolerance test (GTT) (n = 7–8) (**D**) and insulin tolerance test (ITT) (n = 10) at 18 weeks of age. (**F**) Plasma glucose levels at 20 weeks of age (n = 5). Values represent means ± SD.

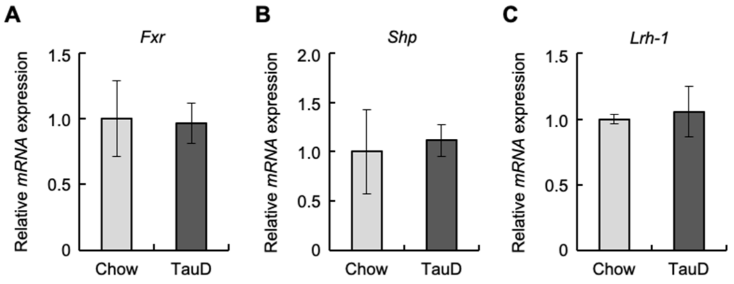

Figure S3. Expression of genes involved in FXR pathway in liver of Chow and TauD groups. The expression of *Fxr* (**A**), *Shp* (**B**), and *Lrh-1* mRNA (**C**) in liver was analyzed by real-time RT-PCR (n = 4). Data were normalized to *Tbp* levels. Values represent means ± SD.

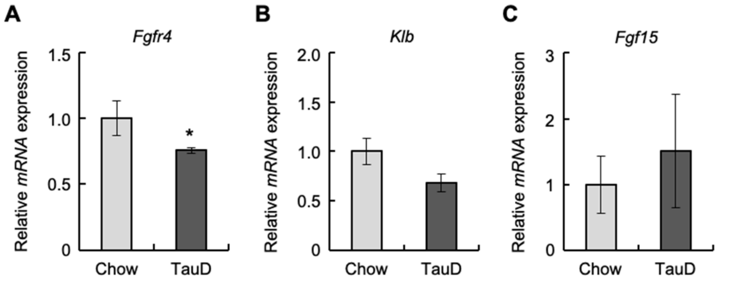

Figure S4. Expression of genes involved in FGFR4/KLB pathway in liver of Chow and TauD groups. The mRNA expression of *Fgfr4* (**A**) and *Klb* (**B**) in liver and *Fgf15* (**C**) in ileum was analyzed by real-time RT-PCR (n = 3–4). Data were normalized to *Tbp* levels. Values represent means ± SD. Differences between values were statistically evaluated by Student's t-test. * $p < 0.05$ vs. Chow.

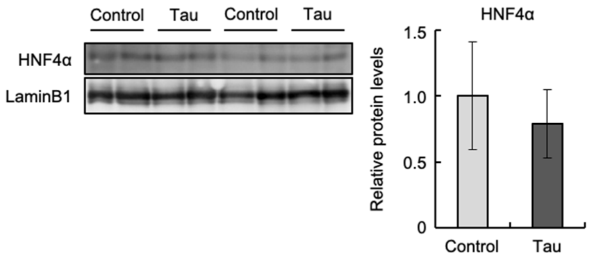

Figure S5. Nuclear HNF4α levels in Hepa1-6 cells. Nuclear protein extracted from 10 mM taurine-treated Hepa1-6 cells was analyzed by immunoblotting using the shown antibodies (n = 4). The left panels show the immunoblotting images. The right graphs show quantitative data. LaminB1 was used as a loading control. Values represent means ± SD.
